# *Lactobacillus rhamnosus* Reduces Blood Glucose Level through Downregulation of Gluconeogenesis Gene Expression in Streptozotocin-Induced Diabetic Rats

**DOI:** 10.1155/2020/6108575

**Published:** 2020-01-13

**Authors:** Eko Farida, Lilis Nuraida, Puspo E. Giriwono, Betty S. L. Jenie

**Affiliations:** ^1^Department of Food Science and Technology, IPB University (Bogor Agricultural University), IPB Dramaga Campus, Bogor 16680, Indonesia; ^2^Department of Nutrition, Faculty of Sport Science, Universitas Negeri Semarang, Sekaran Campus, Gunungpati, Semarang 50229, Indonesia; ^3^Southeast Asian Food and Agricultural Science and Technology (SEAFAST) Centre, IPB University (Bogor Agricultural University), IPB Dramaga Campus, Bogor 16680, Indonesia

## Abstract

Some lactic acid bacteria (LAB) are observed to be potential probiotics with functional properties such as lowering fasting blood glucose (FBG), as a promising hyperglycemia management. This study investigated the ability and mechanism of *Lactobacillus rhamnosus* BSL and *Lactobacillus rhamnosus* R23 on lowering FBG in diabetic rats induced by streptozotocin (STZ). The rats were orally administered with *L. rhamnosus* BSL and *L. rhamnosus* R23 by giving 1 mL cell suspension (10^9^ CFU/mL) daily for 30 days. The body weight (BW) was recorded once in three days, and FBG was recorded once in six days. An oral glucose tolerance test (OGTT) was measured 1 week after injection with STZ and before sacrifice. Fecal samples were collected on days 0, 15, and 30 for LAB population and identification, performed by PCR detecting 16S rRNA. Oral administration of *L. rhamnosus* BSL and *L. rhamnosus* R23 decreased FBG and improved glucose tolerance via downregulation of glucose-6-phosphatase (G6pc) expression by 0.57- and 0.60-fold change, respectively (*P* < 0.05). The lipid profiles, BUN, creatinine, SGOT, and SGPT were significantly (*P* < 0.05) different between normal and diabetic rats, but they were not significantly (*P* > 0.05) different among diabetic rats. Both strains were effective in increasing fecal LAB population. Molecular identification of the isolated LAB from fecal sample indicated that they were able to survive and pass through the digestive tract. These results suggested that both strains have the ability to manage blood glucose level and become a promising agent to manage hyperglycemia and diabetes.

## 1. Introduction

The number of type 2 diabetes mellitus (T2DM) patients has been continuously increasing annually. In 2017, the prevalence of T2DM keeps increasing around the world with the number of 425 million and is predicted to reach 629 million in the year 2045 [1]. Diabetes mellitus is a metabolic disorder characterized by chronic hyperglycemia as a result of damage to insulin production (type 1 diabetes mellitus/T1DM) or insulin resistance (type 2 diabetes mellitus/T2DM) and results in abnormal carbohydrate, fat, and protein metabolism [2].

Insulin resistance in T2DM causes various disruptions on metabolism and regulation processes in the end causing dysfunction of various organs including the kidney and liver. Kidney disorder could be seen from the increase of blood urea nitrogen (BUN) and creatinine [3], while liver dysfunction could be seen from the increase of serum glutamic-oxaloacetic transaminase (SGOT) and serum glutamic-pyruvic transaminase (SGPT) activities [4]. Some studies have indicated that T2DM is often associated with dyslipidemia which is a risk factor causing cardiovascular diseases [5–8]. Recently, probiotic consumption is shown to decrease serum liver enzymes [9, 10]. Previous studies have shown that some probiotic bacteria had the ability to improve serum lipid profiles [11–15]. However, there is limited elucidation on its mechanism.

Hyperglycemia, as observed in T2DM, is associated with increased hepatic glucose production caused by dysregulated gluconeogenesis due to an increased activity of phosphoenolpyruvate carboxykinase (Pepck) and glucose-6-phosphatase (G6pc) [16]. Pepck and G6pc are key enzymes in a gluconeogenesis process. Pepck is an enzyme that catalyzes the synthesis of glucose-6-phosphate from noncarbohydrate precursors, while G6pc catalyzes the dephosphorylation of glucose-6-phosphate to glucose, and this reaction is the last reaction step in gluconeogenesis [17]. Consumption of probiotics has been reported to ameliorate hyperglycemia [18–24]. The probiotic mechanism on hyperglycemia has not yet been fully understood. Some LAB species had been reported as antidiabetic, such as *Lactobacillus reuteri* GMNL-263 [18], *Lactobacillus rhamnosus* CCFM0528 [19], *L. rhamnosus* CCFM0412 [20], *Lactobacillus brevis* and *Lactobacillus plantarum* 13 [21], *L. rhamnosus* NCDC17 [22], and *Lactobacillus casei* CCFM419 [23, 24].

LAB strains isolated from indigenous resources had been tested to have probiotic properties with beneficial function to health. *L. rhamnosus* R23 isolated from human breast milk has antidiarrheal properties [25] and a hypocholesterolemic effect [26]. Research on probiotic utilization had been done considerably; however, the important functional characteristic of that isolate, which is antidiabetic, has not been reported. The ability of LAB to reduce blood glucose is supported by data that both strains are able to survive and pass through the digestive tract. This research was conducted to investigate the ability and clarify the underlying molecular mechanism of *L. rhamnosus* BSL and *L. rhamnosus* R23 on improving blood glucose, serum lipid profile, and renal and liver biomarkers in hyperglycemic rats induced by STZ.

## 2. Materials and Methods

### 2.1. Preparation of Bacterial Cell Suspensions

*L. rhamnosus* BSL and *L. rhamnosus* R23 were obtained from the Food Microbiology Laboratory, Bogor Agricultural University, Indonesia. They were cultured in de Man Rogosa Sharp broth (MRSB) medium (Oxoid, United Kingdom) at 37°C for 18 h. The bacterial cells were collected by centrifugation at 4000 g (4°C) for 10 min to remove the MRSB medium and resuspended in phosphate buffer (Sigma-Aldrich, USA) at a final concentration of 10^9^ CFU/mL.

### 2.2. Animals and Experimental Design

**Figure 1:**
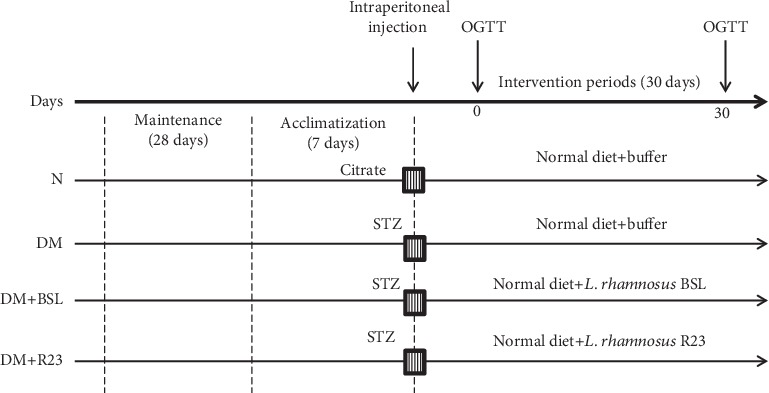
Experimental design.

Animal protocol was approved by the Animal Ethics Committee of Bogor Agricultural University, Indonesia (No. 70-2017 IPB). Male Sprague-Dawley rats (*n* = 24) (body weight (BW) 60-90 g) were obtained from the National Agency for Drug and Food Control, Jakarta, Indonesia. The rats were maintained until BW reached 200-250 g for 28 days and then acclimatized for 7 days. During the acclimatization period, all rats were administered with antibiotics (doxycycline 5 mg/kg BW) and anthelmintic for 3 days. Diabetic rats were prepared by inducing (40 mg/kg BW) with freshly prepared streptozotocin (Sigma-Aldrich, St. Louis, USA) dissolved in 50 mM sodium citrate buffer (pH 4.5), while normal rats received sodium citrate buffer. Hyperglycemia was monitored two times in one week (on the 3^rd^ and 7^th^ days) after injection with STZ and confirmed by fasting blood glucose (FBG) levels higher than 7 mmol/L. The hyperglycemia rats were then divided into three groups (*n* = 6) randomly—DM: diabetic control group (positive control), DM+BSL: STZ group treated by *L. rhamnosus* BSL, and DM+R23: STZ group treated by *L. rhamnosus* R23. Normal rats (N) were used as the negative control. Experimental design is shown in Figure 1.

All rats were given a standard diet (AIN-93M) [27] and provided with water ad libitum. During 30 days of the experimental period, the rats were kept under controlled temperature (22°C ± 3°C). The rats were orally administered with *L. rhamnosus* BSL (DM+BSL group) and *L. rhamnosus* R23 (DM+R23 group) by giving 1 mL of cell suspension (10^9^ CFU/mL) daily for 30 days. The diabetic control group (DM) received 1 mL buffer phosphate. All groups were conducted for an oral glucose tolerance test (OGTT) after 1-week injection with STZ (at day 0) and before sacrifice (at day 30). Fecal samples were collected at days 0, 15, and 30 during the experimental period for total LAB enumeration. The BW of each rat was measured every 3 days. Feed consumption for each rat was determined every day. At the end of experimental periods, rats were fasted overnight and anesthetized by ketamine (80 mg/kg BW) and xylazine (5 mg/kg BW). All rats were sacrificed, and the blood was collected from cardiac puncture for measuring biochemical analysis. The liver organ was collected for measuring Pepck and G6pc gene expressions.

### 2.3. Biochemical Parameter Analysis

Blood samples were centrifuged at 3000 g (5°C) for 15 min to obtain serum. The serum lipid profiles, consisting of total cholesterol (TC), triglyceride (TG), and high-density lipoprotein cholesterol (HDL-c), were quantified using commercial kits (ELITechGroup, France). The low-density lipoprotein (LDL-c) was calculated using Friedewald's equation [28].

(1) $$\begin{array}{c} \text{LDL}-\text{c}=\text{TC}-\text{HDL}-\left(\frac{\text{TG}}{5}\right). \end{array}$$

The atherogenic index (AI) was calculated as follows:

(2) $$\begin{array}{c} \text{AI}=\frac{\left(\text{TC}-\text{HDL}\right)}{\text{HDL}}. \end{array}$$

Serum biomarkers of renal function include blood urea nitrogen (BUN) and creatinine. BUN was determined using BioMaxima SA kit (Lublin, Poland), while creatinine was determined using EliTech kit (France). Serum biomarkers of liver function include glutamic-oxaloacetic transaminase (SGOT) and glutamic-pyruvic transaminase (SGPT) determined using commercial kit (PT Rajawali Nusindo, Indonesia).

### 2.4. Fasting Blood Glucose (FBG) and Oral Glucose Tolerance Test (OGTT)

FBG of each rats was measured once in six days using a glucometer (Allmedicus, Korea) from the tail vein after fasting overnight (approximately 12 h). The OGTT was performed in the beginning and before sacrifice during experimental periods. Rats were orally administered with a glucose solution at a dose of 2 g/kg BW after fasting overnight. Blood glucose from the tail vein was monitored at 0, 15, 30, 60, 90, and 120 min after glucose administration using a glucometer. The glucose response curve was plotted, and the area under the curve (AUC) was calculated using a trapezoidal method.

### 2.5. Analysis of Pepck and G6pc Gene Expression

Total RNA from 30 mg of liver tissue from each rat was extracted using a QIAamp RNA Blood Mini Kit (Qiagen, USA) according to the manufacturer's instructions. The RNA purity and concentration were measured by a NanoDrop 2000 spectrophotometer (Thermo Fisher Scientific, USA). The RNA was then diluted with nuclease-free water to final concentration equivalent to 200 ng/20 *μ*L. The cDNA was synthesized using QuantiTect Reverse Transcription Kit (Qiagen, USA).

Absolute quantification was performed using QuantiTect® SYBR® green PCR kit (Qiagen, USA) and conducted using the RT-qPCR Rotor-Gene Q (Qiagen). The reaction mixture consisted of 12.5 *μ*L QuantiTect SYBR Green master mix, 0.75 *μ*L forward primer (10 *μ*M), 0.75 *μ*L reverse primer (10 *μ*M), and 9.0 *μ*L nuclease-free water which were then homogenized. 2 *μ*L of cDNA sample was added to the reaction mixture and run to PCR as follows: PCR initial activation step at 95°C for 15 min, followed by 40 cycles of denaturation at 94°C for 15 sec, annealing at 59°C for 30 sec and extension at 72°C for 30 sec. An additional final extension at 72°C for 3 sec was included before melting. Primer sequences for the Pepck gene are as follows: forward 5′-GAC AAA TCC GAA CGC CAT TAA G-3′ (ID 99665939) and reverse 5′-TCG ATG CCT TCC CAG TAA AC-3′ (ID 99665940). Primer sequences for the G6pc gene are as follows: forward 5′-CTG GAG TCT TGT CAG GCA TT-3′ (ID 99665937) and reverse 5′-CAG GAA GAA GGT GAT GAC ACA G-3′ (ID 99665938). The standard curve was constructed using serial dilutions of gBlocks®Gene Fragments (ID 99666939) with concentrations of 10^−1^, 10^−2^, 10^−3^, 10^−4^, and 10^−5^ ng/25 *μ*L.

### 2.6. Enumeration of LAB in Fecal Sample Using Plate Count

Fresh fecal samples from three rats per group were collected on days 0, 15, and 30. Each sample was homogenized in sterile buffer solution and made serial dilutions (10^−1^ to 10^−10^). One mL of appropriate dilutions (10^−6^ to 10^−8^) was taken into plates, than poured with MRS agar medium (Oxoid, United Kingdom) supplemented with CaCO_3_ in duplicate. All plates were incubated at 37°C for 48 hours. The number of colony counting for LAB was expressed as log CFU/g fecal.

### 2.7. Isolation and Identification of LAB in Fecal Sample

LAB was isolated from the fecal sample of the DM+BSL and DM+R23 groups. Identification was done to confirm the survival of LAB in the digestive tract. LAB isolates were grown in MRS medium and incubated at 36°C for 24 h. Genomic DNA was extracted using DNA mini kit (Qiagen, USA). The amplification reaction was carried out in a PCR tube containing 1 *μ*L DNA template, 5 *μ*L master mix, 0.25 *μ*L forward primer 63F (5′-CAG GCC TAA CAC ATG CAA GTC-3′), 0.25 *μ*L reverse primer 1387R (5′-GGG CGG AGT GTA CAA GGC-3′), and 3.5 *μ*L of water (dd H_2_O). The amplification by PCR (AB Applied Biosystems, USA) is as follows: initial denaturation (94°C, 5 min, 30 cycles), denaturation (94°C, 30 minutes, 30 cycles), annealing (50°C, 1 min, 30 cycles), extension (72°C, 2 min, 30 cycles), final extension (72°C, 5 min), and cooling (25°C, 10 min). The PCR product was purified using AccuPrep® purification kit (Bioneer, Korea) according the manufacturer's instructions.

The purified PCR product was then sequenced using the Sanger method (ABI 3730 xl DNA analyzer) and analyzed [29]. DNA sequences were edited ,and consensus sequences were obtained using the BioEdit software package. Final sequences were then aligned using Clustal X2 for each of the sequences. The sequences of bacterial isolates of this study were then compared to those in GenBank (National Centre for Biotechnology Information; http://www.ncbi.nih.gov/) using the Basic Local Alignment Search Tool (BLAST). Phylogenetic tree was constructed using the neighbour joining method with MEGA 7.

### 2.8. Statistical Analysis

The data obtained in this experiment were presented as the mean ± standard deviation (SD). Data was analyzed using one-way analysis of variance (ANOVA). The differences between groups were further analyzed using Duncan's multiple range tests. All statistical significance was accepted at *P* value < 0.05. Statistical analysis was performed using SPSS® Statistics software Version 22.

## 3. Results

### 3.1. Effects of *L. rhamnosus* BSL and *L. rhamnosus* R23 on Feed Consumption and Body Weight in Experimental Groups

The daily feed consumption of the N group was significantly (*P* < 0.05) lower than the those of the DM, DM+BSL, and DM+R23 groups, indicating all STZ-treated rats show hyperphagia, a classic indicator of T2DM and hyperglycemia. All rats in the DM, DM+BSL, and DM+R23 groups exhibited significantly (*P* < 0.05) lower final body weight and weight change than the N groups (Table 1). The oral administration of *L. rhamnosus* BSL and *L. rhamnosus* R23 was unable to prevent the decrease of body weight and weight change in the DM+BSL and DM+R23 groups because of insufficient glucose uptake by peripheral tissue; therefore, cellular degradation occurs and is wasting.

### 3.2. Effects of *L. rhamnosus* BSL and *L. rhamnosus* R23 on FBG Changed in Experimental Groups

**Figure 2:**
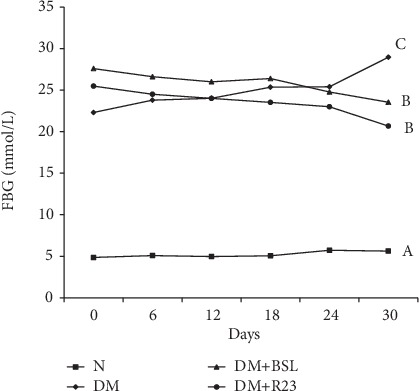
Effect of *L. rhamnosus* BSL and *L. rhamnosus* R23 on fasting blood glucose in experimental groups after intervention period for 30 days. The values are expressed as the means ± SD (*n* = 6). The values with different superscript letters are significantly different (*P* < 0.05).

The FBG in the DM group was significantly higher (*P* < 0.05) than that in the N group during the experimental period. The administration of *L. rhamnosus* BSL and *L. rhamnosus* R23 was observed able to significantly reduce FBG (*P* < 0.05) in the DM+BSL and DM+R23 groups compared to the N and DM groups. However, the decline in FBG was not significantly different (*P* > 0.05) between LAB-treated groups DM+BSL and DM+R23 groups (Figure 2). These data indicated that gavage administration of *L. rhamnosus* BSL and *L. rhamnosus* R23 reduce FBG and can be used for controlling blood glucose in diabetic rats.

### 3.3. Effects of *L. rhamnosus* BSL and *L. rhamnosus* R23 on OGTT in Experimental Groups

**Figure 3:**
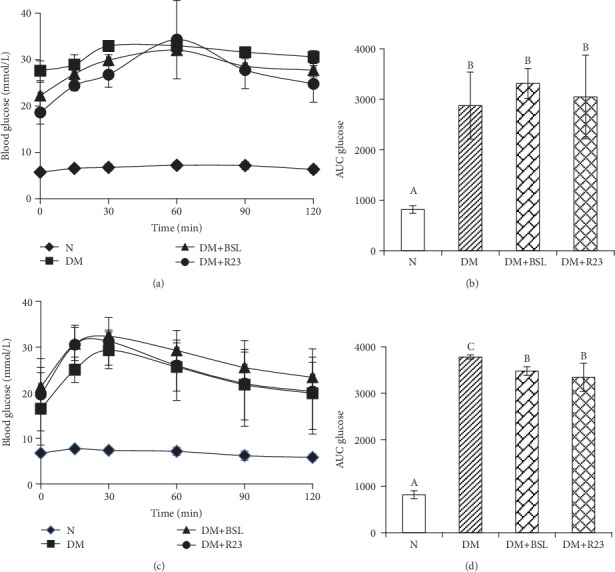
Effect of *L. rhamnosus* BSL and *L. rhamnosus* R23 on OGTT at day 0 (a) and day 30 (c); AUC glucose at day 0 (b) and day 30 (d). The values are expressed as the means ± SD (*n* = 6). The values with different superscript letters are significantly different (*P* < 0.05).

At day 0, glucose tolerance was impaired in the DM, DM+BSL, and DM+R23 groups, which were showed by the AUC glucose values higher than in the N group. There was a significant difference (*P* < 0.05) on AUC glucose values between the N and diabetic groups, but not significantly different (*P* > 0.05) among the diabetic groups (Figure 3(b)). The oral administration of *L. rhamnosus* BSL and *L. rhamnosus* R23 for 30 days was significantly decreased on AUC glucose values (*P* < 0.05) in the DM+BSL and DM+R23 groups compared to the DM group (Figure 3(d)). These data indicate the *L. rhamnosus* BSL and *L. rhamnosus* R23 potential to improve glucose tolerance in diabetic rats.

### 3.4. Effects of *L. rhamnosus* BSL and *L. rhamnosus* R23 on Serum Lipid Profile in Experimental Groups

There was a significant (*P* < 0.05) increase in the serum level of TC, HDL-c, LDL-c, and AI in all diabetic groups compared to the N group (Table 2). *L. rhamnosus* BSL and *L. rhamnosus* R23 administration was able to decrease TC in the diabetic groups. There were no differences in the TG level among the N and diabetic groups during the experimental period (*P* > 0.05). Regarding HDL-c, there were significant (*P* < 0.05) differences among the N and diabetic groups. All diabetic groups showed a significant (*P* < 0.05) increase in HDL-c, but they did not differ significantly from each other.

The administration of *L. rhamnosus* BSL and *L. rhamnosus* R23 had a significant (*P* < 0.05) decrease in the atherogenic index (AI) which is responsible for use as a predictor of cardiovascular diseases (CVD). The present study also showed that *L. rhamnosus* BSL and *L. rhamnosus* R23 played an important role to lower the risk of atherosclerosis by lowering the AI.

### 3.5. Effects of *L. rhamnosus* BSL and *L. rhamnosus* R23 on Renal and Liver Biomarker in Experimental Groups

**Figure 4:**
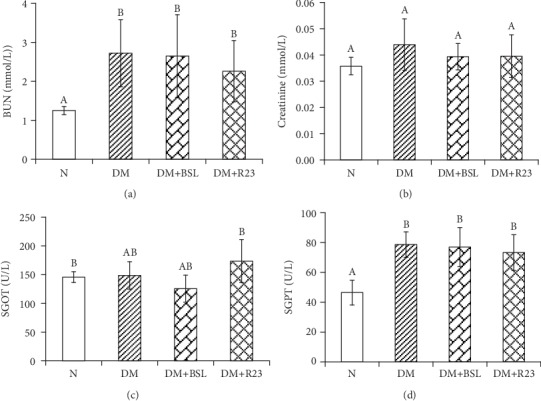
Effect of *L. rhamnosus* BSL and *L. rhamnosus* R23 on renal and liver biomarker in experimental groups after intervention period for 30 days. The values are expressed as the means ± SD (*n* = 6). The values with different superscript letters are significantly different (*P* < 0.05).

Biomarkers of renal function include blood urea nitrogen (BUN) and creatinine. The abnormally high concentrations of BUN and creatinine were consistent with the impaired kidney function. In our present study, there was significant (*P* < 0.05) increase in serum BUN in all diabetic groups when compared to that in the nondiabetic group, but serum creatinine concentration was not different (*P* > 0.05) among the nondiabetic group and diabetic groups (Figure 4).

The effect of *L. rhamnosus* BSL and *L. rhamnosus* R23 administration was observed in serum biomarkers of liver function. In our present study, concentrations of SGOT and SGPT were significantly increased (*P* < 0.05) in diabetic groups compared to those in the N group (Figure 4). Administration of *L. rhamnosus* BSL and *L. rhamnosus* R23 did not significantly (*P* > 0.05) decrease SGOT and SGPT in diabetic groups.

### 3.6. Effects of *L. rhamnosus* BSL and *L. rhamnosus* R23 on Gluconeogenesis Gene Expressions

**Figure 5:**
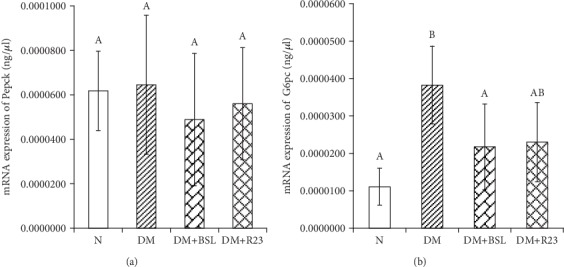
Effect of *L. rhamnosus* BSL and *L. rhamnosus* R23 on Pepck (a) and G6pc (b) mRNA expression in the liver of experimental groups. The values are expressed as the means ± SD (*n* = 6). The values with different superscript letters are significantly different (*P* < 0.05).

The Pepck and G6pc mRNA expression level in the liver are shown in Figure 5. Our results demonstrated that increasing FBG in the diabetic group was caused by increasing expression levels of Pepck and G6pc in diabetic rats. The concentration of Pepck mRNA expression in liver was not different (*P* > 0.05) among the N group and diabetic group, while the G6PC mRNA expression was significantly different (*P* < 0.05) among the N group and diabetic groups (Figure 5). The administration of *L. rhamnosus* BSL and *L. rhamnosus* R23 could not downregulate Pepck gene expression in the liver of diabetic rats. Interestingly, after administration with *L. rhamnosus* BSL and *L. rhamnosus* R23, the expression levels of G6pc decreased almost the same with N group by 0.57- and 0.60-fold changes, respectively (*P* < 0.05). These data indicated that *L. rhamnosus* BSL and *L. rhamnosus* R23 administration downregulates G6pc gene expression, but did not downregulate Pepck gene expression in the liver of diabetic rats.

### 3.7. Effects of *L. rhamnosus* BSL and *L. rhamnosus* R23 on Fecal LAB Population in Experimental Groups

Fresh fecal samples were taken at days 0, 15, and 30 for microbial analysis in order to verify that the strains survived in the gastrointestinal tract. The effectiveness of *L. rhamnosus* BSL and *L. rhamnosus* R23 to reduce FBG was well supported with the increase of fecal LAB population during the experimental period. Fecal LAB population was not significantly different (*P* > 0.05) among the four groups during 15 days of experimental periods. LAB population was significantly increased after 30 days on the diabetic group treated by *L. rhamnosus* BSL and *L. rhamnosus* R23 (Table 3). These results demonstrated that *L. rhamnosus* BSL and *L. rhamnosus* R23 could pass through in the digestive tract and survive detrimental conditions including low pH (in the stomach) and bile salt (in the gut) thus significantly increasing the number of in fecal LAB population on the DM+BSL and DM+R23 groups.

### 3.8. LAB Identification in Fecal Samples of Diabetic Rats

**Figure 6:**
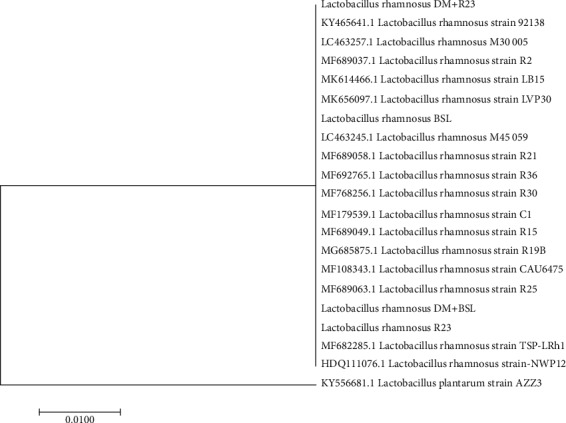
Position of the isolates in the neighbour joining phylogenetic tree based on 16S rRNA gene sequences.

LAB isolates from fecal sample on DM+BSL and DM+R23 were identified using its 16S RNA as *Lactobacillus rhamnosus*. This suggests that both strains were able to tolerate gastric acid and bile salt and survived in the gastrointestinal tract. Phylogenetic tree of LAB isolates in fecal sample is shown in Figure 6.

## 4. Discussion

One novel beneficial effect in probiotics study is their capability to manage blood glucose level [30]. Several animal studies have shown the potency of probiotics in managing T2DM [19, 21, 22, 24, 31]. However, the mechanism is still unclear and thus required further experiment to prove it. In this research, we investigated the effects of indigenous probiotics, namely, *L. rhamnosus* BSL and *L. rhamnosus* R23 administration on lowering blood glucose level in diabetic rats induced by STZ and proposed their possible molecular mechanism through downregulation of gluconeogenesis gene expressions and related parameters. The administration of *L. rhamnosus* BSL and *L. rhamnosus* R23 for 30 days significantly improved hyperglycemia and insulin sensitivity, specifically, in decreasing FBG and improving glucose intolerance, as seen by OGTT. The mRNA expression of gluconeogenesis genes especially G6PC was suppressed after *L. rhamnosus* BSL and *L. rhamnosus* R23 administration.

Diabetic condition usually results in the reduction of body weight although the feed intake is increased [11]. In this present study, the administration of *L. rhamnosus* BSL and *L. rhamnosus* R23 did not prevent weight loss, although the feed intake is increased. Destruction of pancreatic *β*-cells with STZ causes a decrease in *β*-cell integrity and function resulting in a significant decrease of insulin secretion. Glucose as a major energy source is not available in the cell, so the liver will produce glucose from noncarbohydrates such as protein and lipid via gluconeogenesis [16]. Degeneration of tissue lipid and muscle protein for long periods will cause weight loss because they are the major component of body weight.

*L. rhamnosus* BSL and *L. rhamnosus* R23 effectively decreased FBG in the DM+BSL and DM+R23 groups. Our preliminary study demonstrated that ethanol extract from that culture had *α*-glucosidase inhibitory activities by an in vitro study [32]. The inhibition of *α*-glucosidase enzyme could delay the digestion and absorption of carbohydrates and thereby decrease FBG. The *α*-glucosidase enzyme is able to hydrolyze oligosaccharides and disaccharides into glucose/monosaccharides in the small intestine ready for absorption [33]. The ability of probiotics to inhibit *α*-glucosidase may contribute to a decrease in FBG and potentially diabetic prevention and management [33–35]. These findings are in line with other studies that *L. rhamnosus* CCFM0528 [19] and probiotic fermented milk (*L. rhamnosus* MTCC 5957, *L. rhamnosus* MTCC 5897, and *L. fermentum* MTCC 5898) [31] significantly reduced FBG.

The decrease of FBG in the diabetic group treated by *L. rhamnosus* BSL and *L. rhamnosus* R23 was further supported by OGTT as an indicator for T2DM which reflects the function of *β*-cell pancreas to improve glucose tolerance and insulin sensitivity. Our data indicate that both strains improved glucose tolerance and insulin secretion after 30 days of treatment (Figure 5). The administration of *L. rhamnosus* CCFM0528 [19] and *L. rhamnosus* NCDC 17 [22] has been reported to improve glucose tolerance, which is consistent with our results. A further experiment is needed to study the ability of *L. rhamnosus* BSL and *L. rhamnosus* R23 on repairing *β*-cell pancreas regeneration resulting in the improvement of insulin sensitivity.

Lipid metabolism disorder (dyslipidemia) common in T2DM patients causes cardiovascular complication [36]. Dyslipidemia is a lipid metabolism disorder marked with an increasing or decreasing lipid in the blood [37]. The occurrences of dyslipidemia in the society are increasing due to the behavior of consuming low fiber and high-fat level food. Probiotics may improve lipid profile in T2DM [38, 39]. From this study, there was an increase in serum blood lipid profile in the DM, DM+BSL, and DM+R23 groups, and the administration of *L. plantarum* BSL and *L. rhamnosus* R.23 for 30 days was not effective to improve lipid profile, which may be caused by less duration of experimental periods. Interestingly, the atherogenic index in the *L. plantarum* BSL and *L. rhamnosus* R23 groups was significantly decreased to 0.24 and 0.25, respectively, compared to that in the DM group (0.33) and N group (0.22). The administration of *L. rhamnosus* SKG34 and *L. rhamnosus* FBB42 has also decreased the ratios of the atherogenic index [15], which was similar with our results.

In T2DM conditions, kidney and liver disorder is also found, which could be seen from the increase of serum blood urea nitrogen (BUN), creatinine [40], serum glutamic-oxaloacetic transaminase (SGOT), and serum glutamic-pyruvic transaminase (SGPT) concentration [4, 41]. In this study, there was significant increase (*P* < 0.05) in BUN concentration but there was no difference (*P* > 0.05) in creatinine concentration among the normal and diabetic groups (Figure 5). These results are similar with those of a previous study that plasma and urine creatinine were not different between a diabetic group treated with kefir and a diabetic group [42]. The significant increase observed in the BUN concentration of all diabetic groups might be due to the increased synthesis from the damaged pancreatic cells caused by STZ injection. Probiotic bacteria could improve kidney damage by rising gut bacterial metabolism for the excretion of ammonia and reducing BUN concentration [43].

Biomarkers of liver function include SGOT and SGPT, and their measurement is a validated toxicology test [41]. *L. plantarum* BSL had beneficial effects on lowering SGOT and SGPT concentration, while *L. rhamnosus* R23 could affect only SGPT. A previous study showed that administration of multispecies probiotics for 8 weeks among patients with T2DM could be able to lower serum SGPT levels, but did not affect serum SGOT, ALP, and bilirubin levels [44]. Similar with our results, *L. paraplantarum* BGCG11 could decrease serum SGOT and SGPT, indicating that probiotics could improve the condition of the liver in diabetic rats [45]. Another study has shown that soymilk fermented by a mixture of *B. bifidum*, *L. casei*, and *L. plantarum* reduced liver enzymes by decreasing SGPT concentration [33].

The control of blood glucose was greatly affected by glucose production and uptake, and abnormalities of this control cause the progressions of T2DM. Glucose production contributes to hyperglycemia than glucose uptake in T2DM. Glucose was produced through gluconeogenesis and glycogenolysis, but 90% of glucose is obtained from gluconeogenesis, especially in T2DM case [46]. In this study, the disorder of pancreas *β*-cell by STZ induction caused poor insulin secretion and then hyperglycemia occurred. Continuous gluconeogenesis will cause hyperglycemia in long periods [16, 46]. Administration of *L. rhamnosus* BSL and *L. rhamnosus* R23 could decrease serum FBG and may indicate to improve glucose tolerance (Figures 2 and 3). One attempt to explore the mechanism is that we assessed liver mRNA expression of genes involved in gluconeogenesis. G6pc and Pepck are important enzymes in gluconeogenesis which are activated by glucagon and inactivated by insulin [47]. Our results demonstrated that there was a marked increase in Pepck and G6pc gene expression in diabetic compared to normal rats, which was supported in previous results [48]. Administration of *L. rhamnosus* BSL and *L. rhamnosus* R23 could decrease the rate of glucose production and eventual secretion into the blood in LAB-treated diabetic rats by downregulating G6pc expressions and therefore decreasing FBG in the blood. This mechanism was similar with an antidiabetic drug, metformin, which suppressed hepatic glucose production [49]. These results were in line with a previous study that treatment with probiotic fermented milk (*L. rhamnosus* MTCC 5957, *L. rhamnosus* MTCC 5897, and *L. fermentum* MTCC 5898), independently or in combination, has normalized the expression of liver Pepck and G6pc gene expressions [31]. *L. paracasei* TD062 lowered FBG by partially downregulating the expressions of Pepck and G6pc [50], and the same suppression was found by administration with *L. rhamnosus* GG [51], *L. plantarum* MTCC 5690, and *L. fermentum* MTCC 5689 [52].

The colonic bacterial fermentation product, namely, short-chain fatty acid (SCFA), may be key molecules in improving diabetes [53]. SCFA was absorbed into hepatic portal circulation [54, 55] resulting in decreased hepatic gluconeogenesis gene expressions through the AMPK pathway [56]. Propionate might be a substrate for liver gluconeogenesis while acetate and butyrate are involved in liver lipogenesis [57]. *L. plantarum* Ln4 could decrease FBG through the AMPK pathway, especially through phosphorylation of AMPK [58] that led to reduced Pepck and G6pc gene expression [56].

Diabetic condition also changed the intestinal microbiota [59, 60]. Modulation of intestinal microbiota with probiotics may be promising to manage T2DM [61, 62]. In this present study, administration of *L. rhamnosus* BSL and *L. rhamnosus* R23 significantly increased the population of LAB in feces after 30 days of intervention, suggesting that both strains are able to tolerate gastric acid and bile salts and gave their positive effect on managing T2DM through enhancement of beneficial bacteria. This was supported with survival in fecal sample and identified using 16S RNA as *Lactobacillus rhamnosus.* The phylogenetic tree was constructed to saw their relationship with other isolates which were deposited in GenBank (Figure 6). Our results were in line with a previous report that administration of *L. rhamnosus* hsryfm 1301 [63] and *L. rhamnosus* NCDC17 [22] increased the population of lactobacilli significantly than the control group.

**Figure 7:**
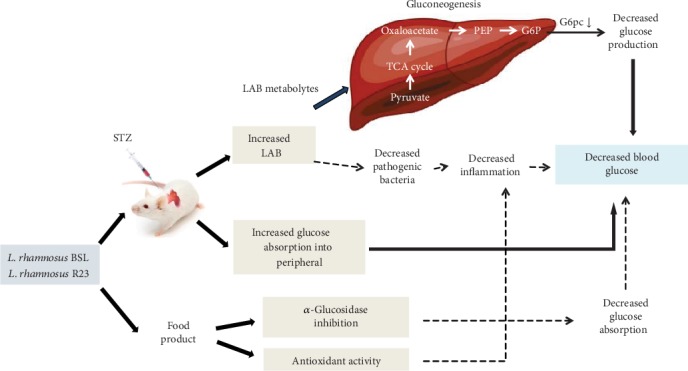
Possible mechanism involved antidiabetic effect of *L. rhamnosus* BSL and *L. rhamnosus* R23 on a diabetic rat model induced by STZ. The alleviation of fasting blood glucose and downregulation of G6pc in diabetic rats was assumed to originate from LAB metabolytes absorbed into the blood and circulated throughout the body.

Some other *Lactobacillus* strains are reported to change the intestinal microbiota and survive in the gastrointestinal tract such as *L. plantarum* 9-41-A and *L. fermentum* M1-16 [64], *L. reuteri* GMNL-263 [18], and *L. casei* CCFM419 [24]. All of the results implicated that probiotic administration has beneficial effects on diabetes through increasing the beneficial bacteria and decreasing the harmful bacteria; however, the exact mechanism of this phenomenon needed further investigation. Based on these results, administration *L. rhamnosus* BSL and *L. rhamnosus* R23 downregulated gluconeogenesis gene expressions, improved glucose intolerance, and therefore reduced FBG. The possible mechanism involved in antidiabetic effects of *L. rhamnosus* BSL and *L. rhamnosus* R23 on a diabetic rat model induced by STZ is provided in Figure 7.

## 5. Conclusions

The administration of *L. rhamnosus* BSL and *L. rhamnosus* R23 for 30 days decreased fasting blood glucose and improved insulin sensitivity through downregulation of glucose-6-phosphatase gen expressions in the liver of a diabetic rat model induced by STZ. Moreover, both strains were also effective in increasing lactic acid bacteria population and survive in fecal sample. These findings suggested that *L. rhamnosus* BSL and *L. rhamnosus* R23 have potential as probiotic foods and become a promising agent to manage T2DM. Further studies on human are required to clarify this antidiabetic effect.

## Figures and Tables

**Table 1:** Feed consumption and body weight in experimental groups.

Parameters	N	DM	DM + BSL	DM + R23
Feed consumption (g/day)	17.07 ± 1.36^a^	19.14 ± 0.79^b^	19.82 ± 0.32^b^	19.15 ± 0.75^b^
Initial body weight (g)	232 ± 15.11^a^	234 ± 12.08^a^	240 ± 18.92^a^	224 ± 23.80^a^
Final body weight (g)	290 ± 15.66^b^	222 ± 11.48^a^	228 ± 16.29^a^	212 ± 23.65^a^
Weight change (g)	(+)58 ± 4.34^b^	(-)12 ± 1.94^a^	(-)12 ± 5.01^a^	(-)12 ± 2.14^a^

The values are expressed as the means ± SD (*n* = 6). Means within a row with different superscript letters are significantly different (*P* < 0.05). N = nondiabetic control group; DM = diabetic control group; DM + BSL = STZ treated with *L. rhamnosus* BSL; DM + R23 = STZ treated with *L. rhamnosus* R23. (+) increased in weight change; (-) decreased in weight change.

**Table 2:** Serum TC, TG, HDL, and LDL cholesterol concentrations in experimental groups.

Lipid profile	N	DM	DM + BSL	DM + R23
Serum TC (mg/dL)	52.17 ± 9.41^a^	79.83 ± 8.16^b^	78.67 ± 10.67^b^	70.83 ± 10.19^b^
Serum TG (mg/dL)	60.17 ± 20.24^a^	79.33 ± 22.95^a^	74.83 ± 24.11^a^	77.17 ± 36.46^a^
HDL-c (mg/dL)	42.83 ± 6.80^a^	60.00 ± 4.05^b^	63.17 ± 4.02^b^	58.67 ± 10.82^b^
LDL-c (mg/dL)	2.70 ± 1.34^a^	5.10 ± 3.29^a^	9.87 ± 5.06^b^	7.13 ± 4.13^ab^
AI	0.22 ± 0.04^a^	0.33 ± 0.10^b^	0.24 ± 0.11^ab^	0.25 ± 0.09^a^

The values are expressed as the means ± SD (*n* = 6). Means within a row with different superscript letters are significantly different (*P* < 0.05). N = nondiabetic control group; DM = diabetic control group; DM + BSL = STZ treated with *L. rhamnosus* BSL; DM + R23 = STZ treated with *L. rhamnosus* R23.

**Table 3:** Fecal LAB populations on days 0, 15, and 30 after *L. rhamnosus* BSL and *L. rhamnosus* R23 administration.

Experimental groups	LAB population (log CFU/g)		
	Day 0	Day 15	Day 30
N	9.52 ± 0.04^a^	9.34 ± 0.05^a^	9.15 ± 0.28^a^
DM	9.53 ± 0.24^b^	9.15 ± 0.10^ab^	8.63 ± 0.03^a^
DM + BSL	9.37 ± 0.02^a^	9.50 ± 0.29^a^	9.68 ± 0.14^b^
DM + R23	9.21 ± 0.05^a^	9.41 ± 0.05^a^	9.80 ± 0.15^b^

The values are expressed as the means ± SD (*n* = 6). Means within a row with different superscript letters are significantly different (*P* < 0.05). N = nondiabetic control group; DM = diabetic control group; DM + BSL = STZ treated with *L. rhamnosus* BSL; DM + R23 = STZ treated with *L. rhamnosus* R23.

## Data Availability

The data used to support the findings of this study are included within the article.
